# Assessment of a PK/PD Target of Continuous Infusion Beta-Lactams Useful for Preventing Microbiological Failure and/or Resistance Development in Critically Ill Patients Affected by Documented Gram-Negative Infections

**DOI:** 10.3390/antibiotics10111311

**Published:** 2021-10-27

**Authors:** Milo Gatti, Pier Giorgio Cojutti, Renato Pascale, Tommaso Tonetti, Cristiana Laici, Alessio Dell’Olio, Antonio Siniscalchi, Maddalena Giannella, Pierluigi Viale, Federico Pea

**Affiliations:** 1 Department of Medical and Surgical Sciences, Alma Mater Studiorum University of Bologna, 40138 Bologna, Italy; milo.gatti2@unibo.it (M.G.); tommaso.tonetti@unibo.it (T.T.); alessio.dellolio@unibo.it (A.D.); maddalena.giannella@unibo.it (M.G.); pierluigi.viale@unibo.it (P.V.); 2 SSD Clinical Pharmacology, Department for Integrated Infectious Risk Management, IRCCS Azienda Ospedaliero-Universitaria di Bologna, 40126 Bologna, Italy; piergiorgio.cojutti@aosp.bo.it; 3 Infectious Diseases Unit, Department for Integrated Infectious Risk Management, IRCCS Azienda Ospedaliero-Universitaria di Bologna, 40126 Bologna, Italy; renato.pascale@aosp.bo.it; 4 Division of Anesthesia and Intensive Care Medicine, IRCCS Azienda Ospedaliero-Universitaria di Bologna, 40126 Bologna, Italy; 5 Division of Anesthesiology, Department of Anesthesia and Intensive Care of Abdominal Organ Transplantation and Hepatobiliary Surgery, IRCCS Azienda Ospedaliero-Universitaria di Bologna, 40126 Bologna, Italy; cristiana.laici@aosp.bo.it (C.L.); antonio.siniscalchi@aosp.bo.it (A.S.)

**Keywords:** PK/PD target attainment, beta-lactams, continuous infusion, critically ill patients, microbiological failure, resistance development, gram-negative infections, C_ss_/MIC, *Pseudomonas aeruginosa*

## Abstract

Background: Emerging data suggest that more aggressive beta-lactam PK/PD targets could minimize the occurrence of microbiological failure and/or resistance development. This study aims to assess whether a PK/PD target threshold of continuous infusion (CI) beta-lactams may be useful in preventing microbiological failure and/or resistance development in critically ill patients affected by documented Gram-negative infections. Methods: Patients admitted to intensive care units from December 2020 to July 2021 receiving continuous infusion beta-lactams for documented Gram-negative infections and having at least one therapeutic drug monitoring in the first 72 h of treatment were included. A receiver operating characteristic (ROC) curve analysis was performed using the ratio between steady-state concentration and minimum inhibitory concentration (C_ss_/MIC) ratio as the test variable and occurrence of microbiological failure as the state variable. Area under the curve (AUC) and 95% confidence interval (CI) were calculated. Independent risk factors for the occurrence of microbiological failure were investigated using logistic regression. Results: Overall, 116 patients were included. Microbiological failure occurred in 26 cases (22.4%). A C_ss_/MIC ratio ≤ 5 was identified as PK/PD target cut-off with sensitivity of 80.8% (CI 60.6–93.4%) and specificity of 90.5% (CI 74.2–94.4%), and with an AUC of 0.868 (95%CI 0.793–0.924; *p* < 0.001). At multivariate regression, independent predictors of microbiological failure were C_ss_/MIC ratio ≤ 5 (odds ratio [OR] 34.54; 95%CI 7.45–160.11; *p* < 0.001) and *Pseudomonas aeruginosa* infection (OR 4.79; 95%CI 1.11–20.79; *p* = 0.036). Conclusions: Early targeting of CI beta-lactams at C_ss_/MIC ratio > 5 during the treatment of documented Gram-negative infections may be helpful in preventing microbiological failure and/or resistance development in critically ill patients.

## 1. Introduction

The global increase of antimicrobial resistance represents a major health concern [1]. Although beta-lactams are still the backbone of treatment for the management of Gram-negative infections in critically ill patients [2], it should be mentioned that the incidence of resistance is rapidly increasing [3].

Beta-lactams exhibit short elimination half-life and time-dependent pharmacodynamics (PD); their efficacy is related to the percentage of the dosing interval in which the free plasma concentration is maintained above the minimum inhibitory concentration (MIC) of the bacterial pathogen (%fT_>MIC_) [4]. Consensus is lacking regarding methods for identifying a pharmacokinetic/pharmacodynamic (PK/PD) target that may maximize the effectiveness of beta-lactams in the treatment of Gram-negative infections among critically ill patients. According to experimental animal models, the minimum threshold needed to achieve bactericidal activity with beta-lactams is 40–70% fT_>MIC_ [5], namely, a target that has been adopted in pivotal trials of novel antimicrobial agents [6,7]. However, emerging clinical data suggest that more aggressive PK/PD targets up to 100%fT_>4–5×MIC_ may give rise to better outcomes in critically ill patients [6,8,9].

Optimizing beta-lactam pharmacodynamics could be a means by which to overcome resistance development [10,11,12]. Preclinical studies with different beta-lactams (namely, piperacillin-tazobactam, meropenem, and ceftazidime) showed that trough concentration (C_min_)/MIC ratios ranging between 3.8 and 6.2 may be helpful in preventing the emergence of resistance among Gram-negatives [13,14,15]. Continuous infusion (CI) may represent the best administration mode for maximizing the pharmacodynamics of beta-lactams under the same daily dose.

The aim of this study was to assess whether a PK/PD target threshold of continuous infusion (CI) beta-lactams may be useful in preventing microbiological failure and/or resistance development in critically ill patients affected by documented Gram-negative infections.

## 2. Results

### 2.1. Patient Population, Microbiological Characteristics, and TREATMENT Regimens

**Figure 1:**
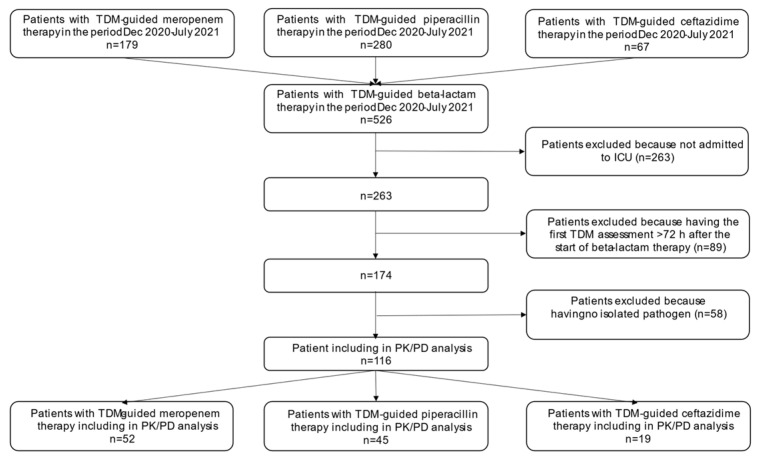
Flowchart of patient inclusion and exclusion criteria. ICU; intensive care unit; PK/PD: pharmacokinetic/pharmacodynamic; TDM: therapeutic drug monitoring.

From December 2020 to July 2021, a total of 526 patients who underwent therapeutic drug monitoring (TDM)-guided beta-lactam therapy in our hospital were screened. Among them, 116 were selected and included in the study (52, 45, and 19 receiving meropenem, piperacillin/tazobactam, and ceftazidime or ceftazidime/avibactam, respectively; see Figure 1). Demographics and clinical characteristics of the included patients are reported in Table 1.

The median age was 66 years (interquartile range [IQR] 56–73 years), and male gender was prevalent (69.8%). The median body mass index (BMI) and median creatinine clearance were 26.3 Kg/m^2^ (IQR 23.5–30.9 Kg/m^2^) and 74.5 mL/min/1.73 m^2^ (IQR 39.8–102 mL/min/1.73 m^2^), respectively. Thirteen out of 116 patients (11.2%) showed augmented renal clearance (ARC) at baseline.

At infection onset, 53.5% of patients had septic shock, 87.1% required mechanical ventilation and 22.4% continuous renal replacement therapy (CRRT).

Nosocomial pneumonia (including both hospital-acquired [HAP] and ventilator-associated pneumonia [VAP]; 49.1%) and bloodstream infections (BSIs; 28.4%) accounted for more than 70% of infections.

Overall, 139 Gram-negative pathogens were isolated. *Klebsiella pneumoniae* was the predominant pathogen (25.2%), followed by *Pseudomonas aeruginosa* (23.7%), *Escherichia coli* (19.4%), *Enterobacter* spp. (10.1%), *Proteus mirabilis* (5.0%), and *Acinetobacter baumannii* (4.3%). Among *Enterobacterales* isolates, 15.5% were extended-spectrum beta-lactamase (ESBL)-producers and 9.3% were *Klebsiella pneumoniae* carbapenemase (KPC)-producers. OXA-48 beta-lactamase was detected in three cases, of which two also coharbored KPC genes. Among *Pseudomonas aeruginosa* isolates, 40.6% were resistant to meropenem with an MIC ≥ 4 mg/L. The MIC ranges of the bacterial clinical isolates are summarized in Supplementary Table S1. Polymicrobial infection occurred in 31 out of 116 cases (22.3%).

Meropenem was the most frequent treatment option (44.8%), followed by piperacillin/tazobactam (38.8%), ceftazidime/avibactam (9.5%), and ceftazidime (6.9%). Combination therapy was used in 19.8% of patients, and included intravenous colistin (n = 9), fosfomycin (n = 8), tigecycline (n = 5), and ciprofloxacin (n = 1). The median duration of beta-lactam treatment was 10 days (IQR 6–14 days). At first TDM assessment, median steady-state concentration (C_ss_) of meropenem, piperacillin, and ceftazidime, were 22.9 mg/L (IQR 14.9–31.6 mg/L), 80.2 mg/L (IQR 56–145 mg/L), and 22.4 mg/L (IQR 15.2–45.5 mg/L) respectively. The median C_ss_/MIC ratio of meropenem, piperacillin, and ceftazidime, were 32.4 (IQR 3.9–211.3), 11.3 (IQR 6.7–19.5), and 21 (IQR 4.5–38), respectively.

### 2.2. Microbiological Failure and Resistance Development

Overall, microbiological failure occurred in 26 patients (22.4%), most of whom (20/26) developed resistance to selected beta-lactams. Demographics and clinical features of critically ill patients who experienced microbiological failure are reported in Supplementary Table S2. Microbiological failure occurred in 13, 10, and 3 patients who were treated with meropenem, piperacillin/tazobactam, and ceftazidime/avibactam, respectively. In 9 out of 26 cases (34.6%), treatment was escalated to combination therapy. Median time to microbiological failure was 11.5 days (IQR 8.3–14 days). Pneumonia accounted for the majority of microbiological failure (65.4%), followed by complicated intrabdominal infections (19.2%) and BSI (15.4%). *Pseudomonas aeruginosa* was the predominant pathogen implicated in microbiological failure (12/26 cases), followed by *Klebsiella pneumoniae* (8 cases, of which three were multisusceptible, two were KPC-producers, one was KPC/OXA-48 coharboring and one each were OXA-48-producing and ESBL-producing), *Acinetobacter baumannii* (4 cases), and *Enterobacter aerogenes* (2 cases). Resistance development occurred in 83.3% and 75% of *Pseudomonas aeruginosa* and *Klebsiella pneumoniae* isolates, respectively.

**Figure 2:**
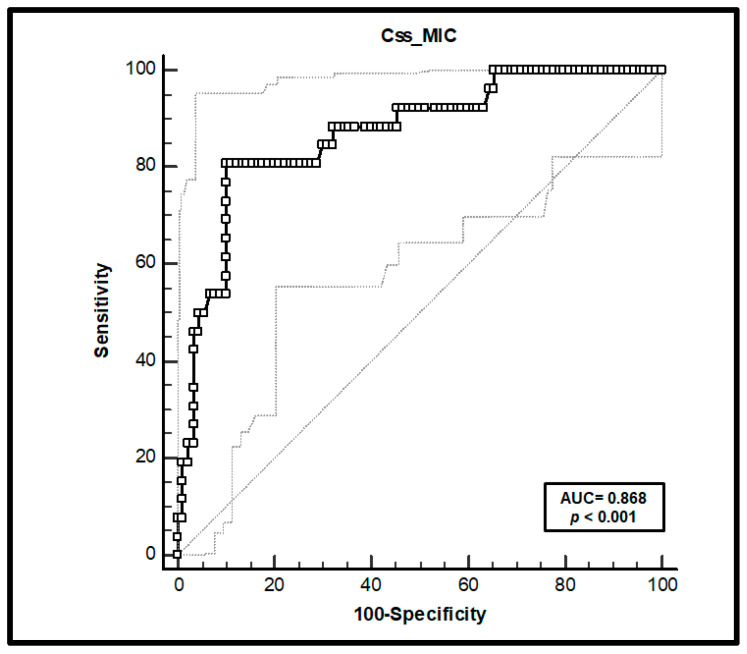
Receiver operating characteristic (ROC) curve analysis. An optimal cut-off of C_ss_/MIC ≤ 5 was found, resulting in a sensitivity of 80.8% and a specificity of 90.0%. The optimal cut-off point was computed using the Youden Index method. AUC: area under the curve; C_ss_: steady-state concentrations.

In the receiver operating characteristic (ROC) analysis, optimal PK/PD target cut-off was identified as C_ss_/MIC ratio ≤ 5, with sensitivity of 80.8% (95% confidence interval [CI] 60.6–93.4%) and specificity of 90.54% (95%CI 81.9–95.3%) (Figure 2), and an area under the curve (AUC) of 0.868 (95%CI 0.793–0.924; *p* < 0.001). The Youden index was 0.71 (95% CI 0.53–0.85).

**Figure 3:**
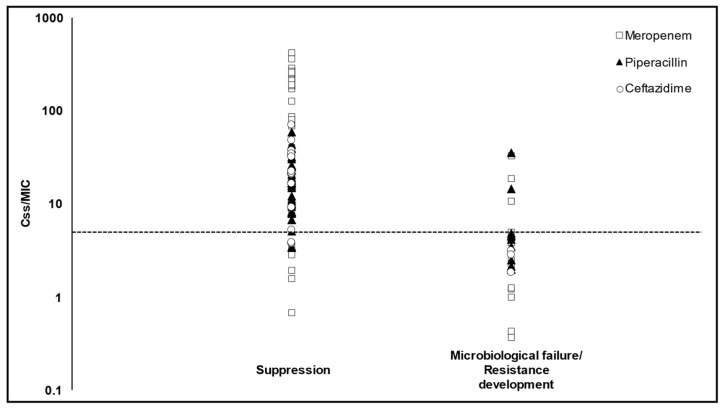
Relationship between beta-lactam PK/PD target attainment (C_ss_/MIC) and microbiological failure and/or resistance development. Each point represents a single critically ill patient treated with selected beta-lactams. Dotted line represents optimal PK/PD cut-off (C_ss_/MIC = 5) identified at ROC analysis. Significantly higher risk of microbiological failure and/or resistance occurrence was found in patients with a C_ss_/MIC below the optimal cut-off (*p* < 0.001). C_ss_: steady-state concentrations.

Significantly higher microbiological failure and/or resistance development was observed in patients with beta-lactam C_ss_/MIC ≤ 5 compared to those with C_ss_/MIC > 5 (21/30 vs. 5/86; *p* < 0.001; Figure 3).

Table 2 summarizes the results of a multivariate regression analysis that assessed possible factors associated with microbiological failure and/or resistance development. C_ss_/MIC ratio ≤ 5 of CI beta-lactams (odds ratio [OR] 34.54; 95%CI 7.45–160.11; *p* < 0.001) and *Pseudomonas aeruginosa* infections (OR 4.79; 95%CI 1.11–20.79; *p* = 0.036) were shown, through multivariate regression analyses, to be independent predictors of microbiological failure and/or resistance development of Gram-negatives among critically ill patients.

## 3. Discussion

To the best of our knowledge, this is the first real-world study that identified significant association between C_ss_/MIC threshold of CI beta-lactams and microbiological failure and/or resistance development among critically ill patients affected by documented severe Gram-negative bacterial infections.

The finding of a C_ss_/MIC ratio ≤ 5 as a strong independent predictor of microbiological failure stresses the relevance that this threshold may have, not only for maximizing clinical efficacy, but also for minimizing the development of resistance [6,10]. This is in agreement with the recommendations of the international guidelines for the management of critically septic patients [9,16]. In our study, it is noteworthy that only a minority (approximatively 5%) of the patients who achieved a PK/PD target above this threshold (namely a 100%T_> 5 × MIC_) within the first 72h experienced microbiological failure or underwent breakthrough resistance.

Our findings are consistent with those of some preclinical models [13,14,15], showing that PK/PD targets required for suppressing the emergence of beta-lactam resistance should be higher compared to those required for clinical efficacy. A dynamic in vitro hollow fiber infection model showed that when using meropenem, ceftazidime, or cefepime in intermittent infusion, a C_min_/MIC ratio > 3.8 may be helpful in suppressing the development of resistance of *Pseudomonas aeruginosa* or *Klebsiella pneumoniae* [13]. In another dynamic in vitro hollow fiber infection model in which intermittent infusion piperacillin–tazobactam was used, it was shown that a C_min_/MIC ratio of 4.6 allowed for resistance suppression when dealing with a relatively low bacterial inoculum of *P. aeruginosa* (4 × 10^5^ CFU/mL) [15]. However, when dealing with a much larger *P. aeruginosa* bacterial load (8 × 10^8^ CFU/mL), the same threshold neither caused any significant bacterial killing nor suppressed the emergence of resistance, [15]. In another dynamic in vitro hollow fiber infection model with intermittent infusion meropenem, it was shown that a C_min_/MIC ratio > 6.2 allowed to suppress resistance development of *Pseudomonas aeruginosa* and that the needed threshold was 4-fold lower when meropenem was combined with aminoglycosides [14]. Consistent with these findings, some authors considered that combination therapy of meropenem plus an aminoglycoside could be helpful for treating Gram-negative infections and for suppressing the emergence of resistance in the presence of a high bacterial burden, as is commonly the case in VAP [11,17]. However, the role of combination therapy is not supported by our analysis, that showed no benefit of combo therapy compared to beta-lactam monotherapy in preventing microbiological failure or resistance development. Conversely, our analysis was in agreement with the findings of a recent retrospective observational multicenter study of ceftazidime/avibactam, which showed no benefit of combination therapy compared to monotherapy [18].

Notably, the achievement of a specific PK/PD threshold with CI compared to intermittent infusion may show remarkable advantages for beta-lactams. The time-dependent PD activity coupled with the short elimination half-lives make this administration mode suitable. Advantages may include administration of lower doses, minimization of fluctuations in antibiotic serum levels, and avoidance of high peak concentrations commonly reported with intermittent infusion and potentially associated with the occurrence of toxicity (e.g., neurotoxicity) [7,19,20].

Infections caused by *Pseudomonas aeruginosa* also emerged as an independent risk factor for microbiological failure and/or for resistance development in this study. It should not be overlooked that in our study, approximately half of the critically ill patients who had microbiological failure and/or resistance development were affected by *Pseudomonas aeruginosa* infection. *Pseudomonas aeruginosa* infection has been identified as a significant predictor of high resistance rate, especially when treatment is based on piperacillin/tazobactam or meropenem [21,22]. The use of extended-infusion (EI) and/or CI may represent an effective strategy for preventing the development of resistance with beta-lactams. Interestingly, EI of ceftolozane/tazobactam, by allowing the achievement of higher PK/PD target compared to intermittent infusion, has emerged as a protective factor in terms of resistance development in patients affected by carbapenem-resistant *Pseudomonas aeruginosa* [23]. Consequently, we believe that in the treatment of critically ill patients affected by *Pseudomonas aeruginosa* infections, the use of high-doses CI beta-lactams, focused on achieving an early, aggressive PK/PD target of C_ss_/MIC > 5, may represent a valuable approach for suppressing resistance development and preventing the emergence of MDR/XDR *Pseudomonas aeruginosa* colonization. This approach may be even more relevant when dealing with challenging pathophysiological conditions (e.g., augmented renal clearance) and/or with deep-seated infections (e.g., pneumonia) [7,24,25]. In this regard, it should not be overlooked that the bacterial burden in pneumonia is usually higher compared to that observed in other sources of infection (e.g., urinary tract infections). This inoculum effect in VAP may attenuate the effectiveness of beta-lactams [19]. Notably, piperacillin-tazobactam and the antipseudomonal cephalosporins were shown to be especially prone to the inoculum effect in *Pseudomonas aeruginosa* infections [26]. Consequently, the identification of more aggressive PK/PD targets is mandatory in VAP, as suboptimal exposure may potentially favor the development of resistance.

Our analysis was limited only to the old beta-lactams, but we are confident that the same principles could be applied to the novel agents (e.g., ceftolozane-tazobactam, ceftazidime-avibactam, meropenem-vaborbactam, cefiderocol) as well. Novel beta-lactams represent the last resort for the management of carbapenem-resistant *Enterobacterales* and/or MDR/XDR *Pseudomonas aeruginosa* or *Acinetobacter baumannii*, and the definition of which PK/PD target threshold should be granted for preventing microbiological failure and for avoiding the development of resistance is one of the major clinical issue that must be addressed in the next few years [11].

We are aware of some limitations in our study. The limited sample size and the retrospective, monocentric study design should be acknowledged. The analysis was based on total beta-lactam concentrations. However, considering the low plasma protein binding (ranging from <10% for meropenem and ceftazidime and approximately 20–30% for piperacillin), no relevant impact on C_ss_/MIC ratio calculation would be expected. The analysis did not take into account the role of beta-lactamase inhibitors (i.e., tazobactam and avibactam). However, it should be mentioned that no definite PK/PD indexes for the beta-lactamase inhibitors were established in preclinical models for resistance suppression. The presence of combination therapy and of polymicrobial infections could be potential confounders, but they occurred only in a minority of cases (<25%). Finally, MIC values were determined by automated testing methods and not through broth microdilution.

In conclusion, this is the first real-world study to have identified a significant association between C_ss_/MIC threshold of CI beta-lactams and microbiological failure and/or resistance development in Gram-negative infections. Both microbiological failure and/or resistance development in Gram-negative infections could be prevented by the early achievement of an aggressive PK/PD target of Css/MIC > 5 during treatment with CI beta-lactams in critically ill patients. Further prospective studies are warranted in order to confirm these findings and identify whether this PK/PD index could be applied to novel beta-lactams as well.

## 4. Materials and Methods

### 4.1. Patients

All the critically ill patients admitted to the general intensive care unit (ICU), transplant ICU, or COVID ICU of the IRCCS Azienda Ospedaliero-Universitaria in Bologna from December 2020 to July 2021 who were treated with beta-lactams because of suspected or documented Gram-negative infections were retrospectively retrieved. Inclusion criteria were: (1) use of piperacillin-tazobactam, ceftazidime, ceftazidime-avibactam, or meropenem by continuous infusion (CI) for at least 72 h; (2) TDM performed in the first 72 h after starting treatment; (3) isolation of Gram-negative pathogens from microbiological cultures and determination of susceptibility for the specific beta-lactam (namely punctual MIC value).

### 4.2. Beta-Lactam Administration and Sampling

Selected beta-lactams were prescribed at the discretion of the treating physician or infectious disease consultant in terms of therapeutic indication, dosage, and duration according to current clinical practice implemented at the IRCCS Azienda Ospedaliero-Universitaria in Bologna. For all the selected beta-lactams, a loading dose (LD), (2 g for meropenem and ceftazidime, 2.5 g for ceftazidime-avibactam and 9 g for piperacillin-tazobactam) was administered over 2-h infusion. Maintenance dose (MD) was administered by CI (q6–8 h infused over 6- and 8-h for meropenem and ceftazidime-avibactam due to stability restrictions; over 24-h for piperacillin-tazobactam and ceftazidime according to stability in aqueous infusion [27]), and dosing regimens were selected at the discretion of the treating physician or infectious disease consultant according to renal function and underlying pathophysiological conditions.

Blood samples were collected in the first 72 h from the beginning of antibiotic treatment in order to determine beta-lactam C_ss_. Total blood concentrations of piperacillin, ceftazidime, and meropenem were measured at the hospital Unique Metropolitan Laboratory concentrations were analyzed by means of a liquid chromatography-tandem mass spectrometry (LC–MS/MS) commercially available method (Chromsystems Instruments & Chemicals GmbH, Munich, Germany) and were provided available for clinical review within 6 h from blood collection.

Combination therapy was defined as the concomitant use with a beta-lactam of other antibiotics active against Gram-negatives (namely aminoglycosides, colistin, fosfomycin, fluoroquinolones, and tigecycline).

### 4.3. Data Collection

Demographic (age, sex, weight, height, body mass index [BMI]) and clinical/laboratory data (need for mechanical ventilation and vasopressors, implementation of continuous renal replacement therapy [CRRT] at baseline, creatinine clearance, presence of augmented renal clearance [ARC], site/type of infection, isolated pathogens, MIC, genetic mechanism of resistance, beta-lactam dosing, C_ss_ at the first TDM assessment, implementation of antibiotic combination therapy, treatment duration, occurrence and timing of relapse, resistance development) were collected for each included patient.

### 4.4. Microbiological and Susceptibility Data

C_ss_/MIC ratio was calculated for each patient at that first TDM assessment that was always performed within 72 h from starting treatment. Gram-negative pathogens were isolated from various infection sites: blood, bronchoalveolar lavage, peritoneal fluid, urine, cerebrospinal fluid, and tissue biopsies. For BAL and urine culture a gram-negative bacterial load ≥10^4^ and ≥10^5^ CFU/mL was considered significant, respectively [28]. Genetic analysis was performed in case of isolation of carbapenem-resistant *Enterobacterales*. Carbapenemase type was determined by multiplex immunochromatographic assay NG test CARBA 5 (NG Biotech, Guipry-Messac, France) for detecting the specific carbapenemase enzyme produced (IMP, VIM, NDM, KPC, OXA-48). In patients having multiple Gram-negative isolates, C_ss_/MIC ratio was calculated using the higher MIC value. The MIC of the identified Gram-negative pathogens was determined by means of E-test methodology, and interpreted according to the European Committee on Antimicrobial Susceptibility Testing (EUCAST) clinical breakpoints.

Microbiological failure was defined as the persistence of the same gram-negative pathogen isolated from index culture after ≥7 days from starting beta-lactam treatment, as previously reported [29]. Resistance development was defined as the increase of the MIC of the clinical isolate beyond the EUCAST clinical breakpoint. Microbiological eradication was defined as the presence of negative cultures in at least two subsequent assessments.

### 4.5. Statistical Analysis

Descriptive statistics were used to describe the patient sample, with continuous data presented as median and IQR, while categorial variables were expressed by count or percentage.

The ROC curve analysis was performed using the C_ss_/MIC ratio as the test variable and emergence of relapse/resistance as the state variable, and AUC along with 95%CI was calculated. The optimal cut-off point was computed using the Youden Index method. Youden Index was calculated according to the following equation: sensitivity (%) + specificity (%) − 100.

Univariate comparisons between patients who experienced microbiological failure and those who did not were performed by the Fisher’s exact test or the Chi-Square test. All the independent covariates with a p value of <0.05 at the univariate analysis were included in a multivariate logistic regression model. A *p* value of <0.05 was considered significant. All statistical analyses were performed with SYSTAT version 13 (SYSTAT Software, Inc., Chicago, IL, USA).

## Figures and Tables

**Table 1:** Demographics and clinical characteristics of critically ill patients receiving continuous infusion beta-lactams for treating documented gram-negative infections.

Demographics and Clinical Variables	Overall Included Patients n = 116
*Patient demographics*	
Age (years)	66 (56–73)
Gender (male/female)	81/35 (69.8/30.2)
Body weight (Kg)	80 (70–90)
Body mass index (Kg/m^2^)	26.3 (23.5–30.9)
Creatinine clearance (mL/min/1.73 m^2^) ^1^	74.5 (39.8–102)
Augmented renal clearance (ARC)	13 (11.2)
*Severity of illness*	
Septic shock ^1^	62 (53.5)
Mechanical ventilation ^1^	101 (87.1)
CRRT ^1^	26 (22.4)
*Indication for beta-lactam use*	
HAP/VAP	57 (49.1)
BSI	33 (28.4)
cUTI	13 (11.2)
cIAI	9 (7.8)
SSTI/NSTI	2 (1.7)
Bone and joint infections	1 (0.9)
Meningitis	1 (0.9)
*Isolated gram-negative pathogens* ^2^	
*Klebsiella pneumoniae*	35 (25.2)
*Pseudomonas aeruginosa*	33 (23.7)
*Escherichia coli*	27 (19.4)
*Enterobacter* spp.	14 (10.1)
*Proteus mirabilis*	7 (5.0)
*Acinetobacter baumannii*	6 (4.3)
*Serratia marcescens*	3 (2.2)
*Others*	14 (10.1)
*Beta-lactam treatment*	
Median meropenem dose (mg/day)	4000 (2000–4000)
Median piperacillin dose (mg/day)	18,000 (18,000–18,000)
Median ceftazidime dose (mg/day)	6000 (6000–6000)
Meropenem C_ss_ (mg/L)	22.9 (14.9–31.6)
Meropenem C_ss_/MIC	32.4 (3.9–211.3)
Piperacillin C_ss_ (mg/L)	80.2 (56–145)
Piperacillin C_ss_/MIC	11.3 (6.7–19.5)
Ceftazidime C_ss_ (mg/L)	22.4 (15.2–45.5)
Ceftazidime C_ss_/MIC	21 (4.5–32.8)
Combination therapy	23 (19.8)
Length of therapy (days)	10 (6–14)
*Clinical outcome*	
Microbiological failure	26 (22.4)
Of which developed resistance	20 (17.2)
Time to microbiological failure (days)	11.5 (8.3–14)

Data are presented as median (IQR) for continuous variables and as n (%) for dichotomous variables. ^1^ At the start of beta-lactam treatment; ^2^ Overall, 139 g-negative pathogens were isolated. BSI: bloodstream infection; cIAI; complicated intrabdominal infection; C_ss_: steady-state concentration; cUTI: complicated urinary tract infection; CRRT: continuous renal replacement therapy; HAP: hospital-acquired pneumonia; IQR: interquartile range; VAP: ventilator-associated pneumonia

**Table 2:** Univariate and multivariate logistic regression analysis of variables associated with microbiological failure and/or resistance development (n = 116).

Variables	Univariate Analysis (OR; 95%CI)	*p* Value	Multivariate Analysis (OR; 95%CI)	*p* Value
*Demographics*				
Age (≥65 years)	1.866 (0.771–4.514)	0.166		
Gender (male)	1.697 (0.914–9.133)	0.071		
Obesity (BMI ≥ 30 kg/m^2^)	0.304 (0.084–1.100)	0.070		
ARC (CL_CR_ ≥ 130 mL/min/1.73 m^2^) ^a^	5.158 (1.556–17.103)	0.007		
*Severity of infection*				
Septic shock ^a^	0.234 (0.089–0.615)	0.003		
Mechanical ventilation ^a^	2.026 (0.427–9.618)	0.374		
CRRT ^a^	1.050 (0.372–2.967)	0.920		
*Type of infection*				
Pneumonia	2.942 (1.159–7.467)	0.023		
BSI	0.382 (0.121–1.212)	0.102		
cIAI	3.091 (0.765–12.483)	0.113		
*Gram-negative isolates*				
*Pseudomonas aeruginosa*	4.360 (1.733–10.966)	0.002	4.79 (1.11–20.79)	0.036
*Klebsiella pneumoniae*	1.303 (0.515–3.295)	0.576		
*Escherichia coli*	0.217 (0.048–0.985)	0.048		
*Enterobacter* spp.	0.542 (0.113–2.591)	0.443		
*Proteus mirabilis*	0.680 (0.076–6.093)	0.730		
*Acinetobacter baumannii*	8.000 (1.376–46.523)	0.021		
*Treatment characteristics*				
C_ss_/MIC ≤ 5	37.800 (11.454–124.741)	<0.001	34.54 (7.45–160.11)	<0.001
Combination therapy	2.874 (1.069–7.725)	0.036		
Treatment duration > 7 days	2.550 (0.880–7.392)	0.085		

Adjusted R^2^ = 0.611; ^a^ At baseline; ARC: augmented renal clearance; BMI: body mass index; BSI: bloodstream infection; cIAI: complicated intrabdominal infection; CI: confidence interval; CL_CR_: creatinine clearance; CRRT: continuous renal replacement therapy; C_ss_: steady-state concentration; MIC: minimum inhibitory concentration; OR: odds ratio.

## Data Availability

The data presented in this study are available on request from the corresponding author. The data are not publicly available due to privacy concerns.

## Supplementary Materials

The following are available online at https://www.mdpi.com/article/10.3390/antibiotics10111311/s1, Table S1: MIC range of gram-negative pathogens (n = 139 from 116 patients) isolated from patients included in the pharmacokinetic/pharmacodynamic analysis. Table S2: Demographics and clinical features of patients with Gram-negative infections showing microbiological failure or resistance development.
