# Supplementary material for: Assessment of a PK/PD Target of Continuous Infusion Beta-Lactams Useful for Preventing Microbiological Failure and/or Resistance Development in Critically Ill Patients Affected by Documented Gram-Negative Infections
Source: Antibiotics (Basel). 2021 Oct 27;10(11):1311. doi: 10.3390/antibiotics10111311 (PMC8615220; doi:10.3390/antibiotics10111311)
Supplement: Supplementary file 1 [file antibiotics-10-01311-s001.zip › antibiotics-1424715-supplementary.pdf]

Table S1. MIC range of gram-negative pathogens (n=139 from 116 patients) isolated from patients included in the pharmacokinetic/pharmacodynamic analysis.

Pathogen	No. of Isolates	MIC Range (mg/L)
	**Meropenem**	
Klebsiella pneumoniae	17	0.12–16
Pseudomonas aeruginosa	16	0.12–8
Escherichia coli	9	0.12–1
Acinetobacter baumannii	6	0.12–32
Proteus mirabilis	3	0.12
Enterobacter aerogenes	3	0.12–0.25
Enterobacter cloacae	2	0.12
Enterobacter absuriae	1	0.25
Serratia marcescens	1	0.12
Hafnia alvei	1	0.12
Klebsiella variicola	1	0.12
Klebsiella oxytoca	1	0.12
Citrobacter freundii	1	0.12
Morganella morgannii	1	0.12
	**Ceftazidime**	
Pseudomonas aeruginosa	2	1–2
Escherichia coli	2	1
Klebsiella pneumoniae	2	1
Klebsiella oxytoca	1	1
Proteus mirabilis	1	1
Enterobacter aerogenes	1	1
**Ceftazidime-Avibactam**		
Klebsiella pneumoniae	7	2–4
Pseudomonas aeruginosa	3	4–8
Enterobacter aerogenes	1	2
	**Piperacillin-Tazobactam**	
Escherichia coli	16	4–16
Pseudomonas aeruginosa	12	4–16
Klebsiella pneumoniae	9	8–16
Enterobacter aerogenes	5	8–16

Proteus mirabilis	3	8
Klebsiella oxytoca	3	8
Serratia marcescens	2	8–16
Citrobacter koseri	2	8
Enterobacter cloacae	1	8
Klebsiella variicola	1	8
Providencia stuartii	1	8
Prevotella buccae	1	4

MIC: minimum inhibitory concentration.

Table S2. Demographics and clinical features of patients with Gram-negative infections showing microbiological failure and/or resistance development.

Case ID	Age/Sex	Antibiotic Therapy/ Dosage	Combination Therapy	Type of Infection	Isolates/MIC (Index Cultures)	C_{ss}/MIC	Latency for Microbiological Failure (Days)	Isolates/MIC (Relapse)	Outcome
#1	51/M	Meropenem 500 mg q6h over 6h infusion	No	VAP	*Pseudomonas aeruginosa* – 8 mg/L	3.84	14	*Pseudomonas aeruginosa* – 32 mg/L (VAP)	Recovered
#2	66/M	Meropenem 1 g q6h over 6h infusion	Fosfomycin	VAP	*Pseudomonas aeruginosa* – 8 mg/L	1	8	*Pseudomonas aeruginosa* – 8 mg/L (BSI)	Died
#3	31/M	Meropenem 1 g q6h over 6h infusion	Fosfomycin + Tigecycline	cIAI	*Acinetobacter baumannii* – 32 mg/L	1.25	13	*Acinetobacter baumannii* 32 mg/L (cIAI)	Died
#4	36/F	Meropenem 500 mg q6h over 6h infusion	No	BSI/VAP	*Pseudomonas aeruginosa* – 1 mg/L	2.5	7	*Pseudomonas aeruginosa* – 8 mg/L (VAP)	Recovered
#5	55/F	Meropenem 1 g q6h over 6h infusion	Colistin	VAP	*Acinetobacter baumannii* – 32 mg/L	1.23	9	*Acinetobacter baumannii* – >32 mg/L (VAP)	Recovered
#6	56/M	Meropenem 1 g q6h over 6h infusion	Colistin	VAP	*Acinetobacter baumannii* – 32 mg/L	0.44	14	*Acinetobacter baumannii* – 32 mg/L (VAP)	Died
#7	66/F	Meropenem 500 mg q6h over 6h infusion	No	CR-BSI	ESBL-producing Kp – 1 mg/L	18.9	14	ESBL-producing Kp – 1 mg/L (CR-BSI)	Died
#8	52/M	Meropenem 1 g q6h over 6h infusion	No	VAP	*Pseudomonas aeruginosa* – 1 mg/L	33.1	12	*Pseudomonas aeruginosa* – 32 mg/L (VAP)	Recovered
#9	84/M	Meropenem 500 mg q8h over 8h infusion	No	cIAI	*Enterobacter aerogenes* – 0.25 mg/L	10.8	10	*Enterobacter aerogenes* – 2 mg/L (cIAI)*	Recovered

#10	74/M	Meropenem 500 mg q6h over 6h infusion	Tigecycline	cIAI	*Acinetobacter baumannii* – 32 mg/L	0.37	7	*Acinetobacter baumannii* – >32 mg/L (cIAI)	Died
#11	73/M	Meropenem 250 mg q6h over 6h infusion	No	VAP	*Pseudomonas aeruginosa* – 4 mg/L	3.88	12	*Pseudomonas aeruginosa* – 8 mg/L (VAP)	Died
#12	64/M	Meropenem 1 g q6h over 6h infusion	Colistin	BSI/VAP	OXA-48-producing Kp – 16 mg/L	2.19	25	OXA-48-producing Kp – 32 mg/L (VAP)	Recovered
#13	15/M	Meropenem 1 g q6h over 6h infusion	Fosfomycin + Tigecycline	VAP	*Pseudomonas aeruginosa* – 1 mg/L	5	19	*Pseudomonas aeruginosa* – 16 mg/L (VAP)	Recovered
#14	77/M	Ceftazidime-avibactam 2.5 g q8h over 8h infusion	Tigecycline	cIAI/BSI	KPC-producing Kp – 4 mg/L	2.85	10	*Klebsiella pneumoniae* KPC – 64 mg/L (VAP)	Recovered
#15	16/M	Ceftazidime-avibactam 2.5 g q8h over 8h infusion	No	VAP	KPC-producing Kp – 2 mg/L	3.15	8	*Klebsiella pneumoniae* KPC – 4 mg/L (VAP)	Recovered
#16	46/M	Ceftazidime-avibactam 2.5 g q8h over 8h infusion	No	VAP	KPC/OXA-48-producing Kp – 4 mg/L	1.85	15/31	KPC/OXA-48-producing Kp – >16 mg/L (CR-BSI)	Recovered
#17	76/F	Piperacillin-tazobactam 18 g/day CI	No	VAP	*Enterobacter aerogenes* – 8 mg/L	14.63	15	ESBL-producing *Enterobacter aerogenes* – 16 mg/L (BSI)*	Recovered
#18	65/M	Piperacillin-tazobactam 18 g/day CI	No	VAP	Kp – 8 mg/L	3.5	8	Kp – 8 mg/L (VAP)	Recovered
#19	60/M	Piperacillin-tazobactam 18 g/day CI	No	VAP	*Pseudomonas aeruginosa* – 8 mg/L	4.63	8	*Pseudomonas aeruginosa* – >16 mg/L (VAP)	Died

#20	16/M	Piperacillin-tazobactam 13.5 g/day CI	No	cIAI/VAP	Kp – 8 mg/L	2.5	7	Kp - >128 mg/L (VAP)	Recovered
#21	37/M	Piperacillin-tazobactam 9 g/day CI	No	VAP	Kp – 16 mg/L	2.02	10	Kp - >16 mg/L (VAP)	Recovered
#22	72/M	Piperacillin-tazobactam 18 g/day CI	No	BSI	*Pseudomonas aeruginosa* – 8 mg/L	4.46	28	*Pseudomonas aeruginosa* – >16 mg/L (cUTI)	Recovered
#23	73/M	Piperacillin-tazobactam 18 g/day CI	No	VAP	*Pseudomonas aeruginosa* – 8 mg/L	36	11	*Pseudomonas aeruginosa* – >16 mg/L (VAP)	Died
#24	80/M	Piperacillin-tazobactam 18 g/day CI	No	VAP	*Pseudomonas aeruginosa* – 8 mg/L	4.83	14	*Pseudomonas aeruginosa* – 8 mg/L (VAP)	Recovered
#25	15/M	Piperacillin-tazobactam 18 g/day CI	Fosfomycin	VAP	*Pseudomonas aeruginosa* – 8 mg/L	2.16	9	*Pseudomonas aeruginosa* >16 mg/L (VAP)	Recovered
#26	15/M	Piperacillin-tazobactam 22.5 g/day CI	Fosfomycin + Colistin	VAP	*Pseudomonas aeruginosa* – 16 mg/L	4.17	20	*Pseudomonas aeruginosa* >16 mg/L (VAP)	Recovered

* reduced susceptibility to carbapenems; BSI: bloodstream infection; cIAI; complicated intrabdominal infection; CI: continuous infusion; CR-BSI: catheter-related bloodstream infection; Css: concentration at steady state; cUTI: complicated urinary tract infection; ESBL: extended-spectrum beta-lactamase; ICU: intensive care unit; KPC: Klebsiella pneumoniae carbapenemase; Kp: Klebsiella pneumoniae; MIC: minimum inhibitory concentration; VAP: ventilator-associated pneumonia.
